# Controlled and Efficient Polymerization of Conjugated Polar Alkenes by Lewis Pairs Based on Sterically Hindered Aryloxide-Substituted Alkylaluminum

**DOI:** 10.3390/molecules23020442

**Published:** 2018-02-17

**Authors:** Xiaojun Wang, Yixin Zhang, Miao Hong

**Affiliations:** State Key Laboratory of Organometallic Chemistry, Center for Excellence in Molecular Synthesis, Shanghai Institute of Organic Chemistry, University of Chinese Academy of Sciences, Chinese Academy of Sciences, 345 Lingling Road, Shanghai 200032, China; wangxiaojun@sioc.ac.cn (X.W.); zhangyixin1@sioc.ac.cn (Y.Z.)

**Keywords:** frustrated Lewis pair, Lewis pair, polymerization, carbenes, acrylics

## Abstract

Reported herein is the development of an effective strategy for controlled and efficient Lewis pair polymerization of conjugated polar alkenes, including methyl methacrylate (MMA), *n*-butyl methacrylate (*^n^*BuMA), and γ-methyl-α-methylene-γ-butyrolactone (γMMBL), by the utilization of sterically encumbered Al(BHT)_2_Me (BHT: 2,6-di-*tert*-butyl-4-methylphenol) as a Lewis acid that shuts down intramolecular backbiting termination. In combination with a selected *N*-heterocyclic carbene (NHC) as a Lewis base, the polymerization of MMA exhibited activity up to 3000 h^−1^ TOF and an acceptable initiation efficiency of 60.6%, producing polymers with high molecular weight (*M*_n_ up to 130 kg/mol) and extremely narrow dispersity (*Đ* = 1.06~1.13). This controlled polymerization with a living characteristic has been evidenced by chain-extension experiments and chain-end analysis, and enabled the synthesis of well-defined diblock copolymers.

## 1. Introduction

**Scheme 1:**
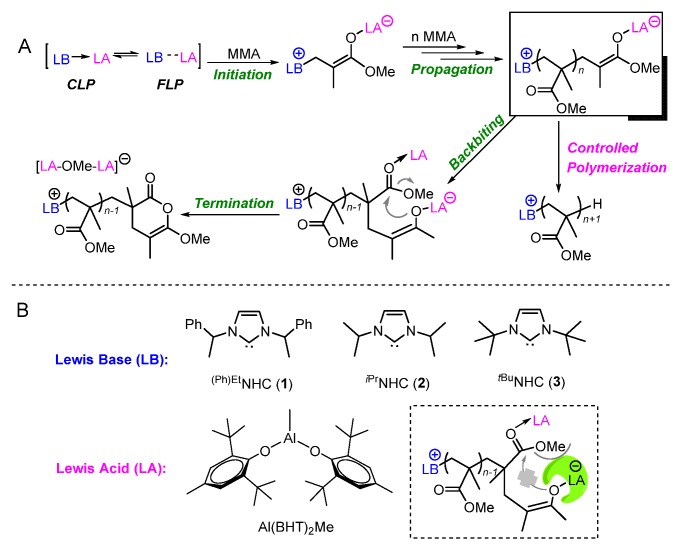
(**A**) Generalized chain initiation, propagation, and termination mechanisms of the Lewis pair polymerization (LPP) of methyl methacrylate (MMA) through zwitterionic intermediates. (**B**) The structures of Lewis acids (LAs) and Lewis bases (LBs) examined in this study.

Lewis pair polymerization (LPP), catalyzed by frustrated Lewis pairs (FLPs) [1,2,3,4,5] or classical Lewis pairs (CLPs) in which Lewis acid (LA) and Lewis base (LB) are involved in monomer substrate activation, chain initiation, as well as chain propagation and termination/transfer steps (Scheme 1A) [6], has recently emerged and attracted enormous interest because the cooperation of a Lewis pair (LP) allows for polymerization that is hardly accessible using a single catalyst and enhances the activity and selectivity of this polymerization. The pioneering work of LPP was first reported by Chen et al. in 2010 utilizing FLPs or CLPs consisting of bulky phosphine or an *N*-heterocyclic carbene (NHC) LB and the highly acidic Al(C_6_F_5_)_3_ LA, which are capable of promoting rapid polymerization of conjugated polar alkenes, such as methyl methacrylate (MMA) and biorenewable γMMBL (turnover frequency (TOF) [7] up to 48,000 h^−1^) [8]. In their latter contributions, this unique catalytic system was extended to the polymerization of other acrylate and acrylamide derivatives [9] as well as monomers with C=C–C=N functionality such as 2-vinyl pyridine and 2-isopropenyl-2-oxazoline [10]. Recently, the highly active LPP of γMMBL by metal-free B(C_6_F_5_)_3_-based LA in combination with NHC or phosphine LB was investigated by Chen et al*.*, who found that interacting LPs or even CLPs are essential for highly effective polymerization, whereas the B/P FLP systems exhibit no or negligible polymerization reactivity [11,12,13]. In 2014, Lu and colleagues reported the chemoselective polymerization of dissymmetric divinyl acrylic monomers by Al(C_6_F_5_)_3_-based CLPs, affording soluble polymers with high molecular weight for post-functionalization [14]. Very recently, Xu and coworkers developed intramolecular and intermolecular rare-earth (RE = Sc, Y, Lu, La)/phosphine LPs for the polymerization of MMA, γMMBL, and other acrylate derivatives [15,16]. Besides the conjugated-addition polymerization of polar alkenes, controlled ring-opening (co)polymerizations of heterocyclic monomers such as lactide, lactones, and α-amino acid *N*-carboxy-anhydride, as well as the alternating ring-opening copolymerization of epoxides with carbonyl sulfide or cyclic anhydrides, have been achieved with BEt_3_, B(C_6_F_5_)_3_, Al(C_6_F_5_)_3_ and Zn(C_6_F_5_)_2_-based LPs [17,18,19,20,21,22,23,24,25].

Controlled polymerization with a living characteristic is a powerful strategy for the precise control of macromolecular structure, which enables the addition of functionality to materials for practical applications [26,27,28,29,30,31,32]. Though exciting results have been achieved in the LPPs of conjugated polar alkenes, these polymerizations were unfortunately difficult to control, presumably due to undesired chain termination proceeding via intramolecular backbiting to generate a six-membered lactone (Scheme 1A) [33,34,35]. To avoid chain termination, the strategy reported in the literatures has been the utilization of weak acidic LAs, which weakens the activation of adjacent ester groups of the growing polymer chain, thereby suppressing the backbiting reaction. However, the controlled LPP via this strategy is usually at the expense of polymerization activity, as the weak acidic LA also impairs the activation of monomers, thus leading to slow propagation. For example, Rieger and co-workers reported a controlled LPP of *N*,*N*-dimethylacrylamide (DMAA) and methylacrylate derivatives, such as furfuryl methacrylate (FMA), *^n^*BuMA, and *t*-butyl methacrylate (*^t^*BuMA), via highly interacting CLPs composed of weak acidic organoaluminum LAs (AlMe_3_ and AlEt_3_) and less sterically hindered phosphines (PMe_3_ and PEt_3_), but the polymerization activity was relatively low (TOF: 67~500 h^−1^) [36]. Likewise, Taton et al. established a controlled but low active polymerization of MMA (TOF < 14.3 h^−1^) via metal-free LPs composed of a weak silane LA (Me_3_SiNTf_2_, Me_3_SiOTf) and phosphine LB [37].

As shown by the above overview, the development of an effective LPP of conjugated polar alkenes that not only exhibits good activity but also has a high degree of control over the polymerization has remained elusive and is the central goal of this study. In this context, we set out to examine the LPPs toward three representative acrylic monomers, including MMA,*^n^*BuMA, and γMMBL using Al(BHT)_2_Me as an LA (Scheme 1B), which is more sterically hindered but slightly less Lewis-acidic compared to commonly used Al(C_6_F_5_)_3_. We envisioned that the introduction of such a sterically encumbered LA would block intramolecular backbiting termination (Scheme 1B); furthermore, we figured that the LA’s suitable Lewis acidity as well as the utilization of a strong nucleophile NHC (^(Ph)Et^NHC, *^i^*^Pr^NHC, and *^t^*^Bu^NHC) would ensure good activity and thus would present an opportunity for efficient and controlled polymerization.

## 2. Results and Discussion

### 2.1. Interaction of Al(BHT)_2_Me with ^(Ph)Et^NHC

The reactivity of the selected NHC (^(Ph)Et^NHC) toward equivalent Al(BHT)_2_Me was examined at the NMR scale in J-Young NMR tube using bezene-d_8_ as the solvent at room temperature (RT). ^1^H-NMR analysis revealed that the reaction between ^(Ph)Et^NHC and Al(BHT)_2_Me was rapid, which was completed in 10 min, affording a clean ^(Ph)Et^NHC→Al(BHT)_2_Me CLP adduct. The characteristic signals corresponding to the >CHCH_3_ group of the ^(Ph)Et^NHC were downfield-shifted by around 0.6 ppm after the reaction, which is indicative of the strong donation of electron density from the carbene center to the Lewis-acidic Al center. Interestingly, two sets of signals can be observed for the >CHCH_3_ and –CH=CH– groups of ^(Ph)Et^NHC with a ratio of around 6:4, indicating that the resultant adduct contains two isomers, presumably caused by the constrained geometry of ^(Ph)Et^NHC in the adduct due to the sterically hindered Al(BHT)_2_Me. The stoichiometric reaction of Al(BHT)_2_Me with MMA was also investigated in the bezene-d_8_ by ^1^H-NMR. Most significantly, the signals attributed to the MMA shifted upfield (=CH_2_: 5.11 ppm, –OCH_3_: 3.17 ppm, –CH_3_: 1.39 ppm) (exception: =CH_2_: 6.20 ppm) relative to free MMA (=CH_2_: 6.07 and 5.19 ppm, –OCH_3_: 3.36 ppm, –CH_3_: 1.80 ppm), suggesting the formation of MMA→Al(BHT)_2_Me CLP adduct and the ability of Al(BHT)_2_Me to activate MMA. In contrast, the signals of MMA was shifted upfield even further (=CH_2_: 5.85 and 4.98 ppm, –OCH_3_: 3.11 ppm, –CH_3_: 1.30 ppm) when coupled with Al(C_6_F_5_)_3_ [8], which is consistent with the fact that the Lewis acidity of Al(BHT)_2_Me is weaker than that of Al(C_6_F_5_)_3_. We also tried to detect the zwitterionic imidazolium enolaluminate intermediate by the reaction of MMA→Al(BHT)_2_Me with ^(Ph)Et^NHC at RT in bezene-d_8_. However, ^1^H-NMR spectrum after the reaction was very complex and the signals attributed to zwitterionic imidazolium enolaluminate were undetectable, though the signals corresponding to MMA→Al(BHT)_2_Me and ^(Ph)Et^NHC vanished, presumably a result of the limited stability of zwitterionic intermediate at RT in the absence of a monomers.

### 2.2. Characteristic of Polymerization by NHC/Al(BHT)_2_Me

**Figure 1:**
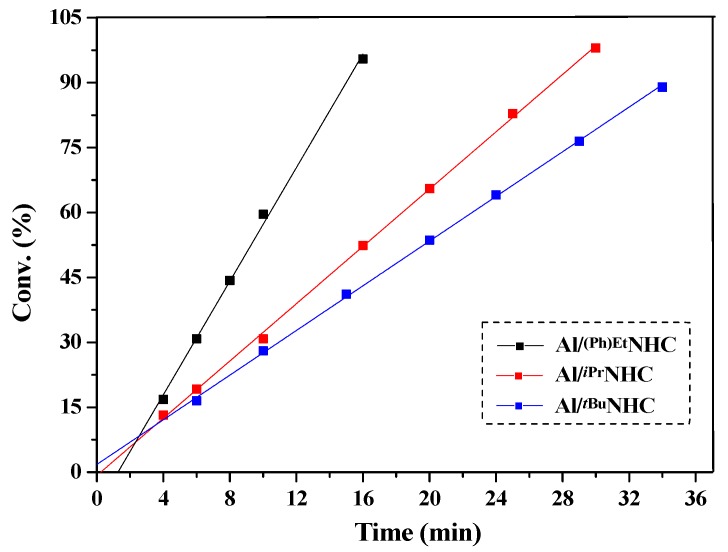
Conversion–time plots for the MMA polymerization by different Lewis pairs (LPs) in toluene at room temperature (RT).

**Figure 2:**
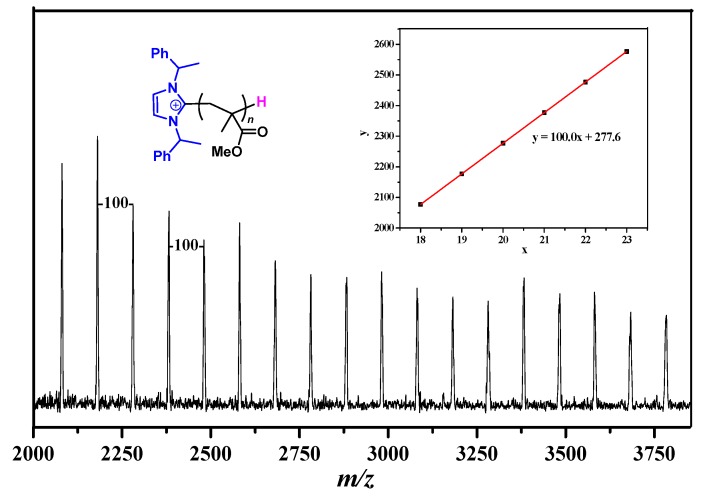
MALDI-TOF MS analysis for PMMA oligomers produced by Al(BHT)_2_Me/^(Ph)Et^NHC in toluene at RT. Inset: a plot of *m*/*z* values (y) vs. the number of MMA repeat units (x).

The initial investigation was carried out using an Al(BHT)_2_Me LA or ^(Ph)Et^NHC LB alone for polymerization of MMA at RT in toluene, and these control runs yielded no polymer formation for up to 24 h (Table 1, Runs 1 and 2). Intriguingly, the polymerization became highly active when premixing Al(BHT)_2_Me with MMA in toluene followed by the addition of ^(Ph)Et^NHC to start the polymerization, which can convert all monomers into polymers in 3 min (TOF = 2000 h^−1^, Table 1, Run 3), which is consistent with the bimolecular, activated monomer mechanism proposed for LPPs in the literature. In sharp contrast, no polymerization was observed when the Al(BHT)_2_Me LA was coupled with P*^t^*Bu_3_ LB [9], demonstrating that the cooperation of LA and LB components is critical to the reactivity of LPPs. Note that the dispersity of the obtained PMMA was very narrow (*Đ* = 1.11), despite the fact that the resulting *M*_n_ (54.1 kg/mol) was higher than the theoretical value. The low initiation efficiency (I^*^ = 19.0%) was presumably caused by the stable NHC/Al adduct, which is difficult to dissociate in the presence of monomers so as to generate free NHC for initiating polymerization. A reversed addition sequence by premixing Al(BHT)_2_Me and ^(Ph)Et^NHC first and then adding MMA resulted in low active polymerization (TOF = 200 h^−1^, Table 1, Run 4) with an I^*^ of only 3.0%, indicating the importance of preventing the contact of Al(BHT)_2_Me and NHC before initiation. Nevertheless, the problem of low I^*^ can be solved by decreasing the catalyst loading to 0.2 mol %, which leads to polymerization with an I^*^ of 50.0% (Table 1, Run 5). As shown in Figure 1, monomer conversion increases linearly with polymerization time after an initial induction period (1.2 min). The quantitative conversion can be achieved in 16 min, affording the polymer a TOF value of 1875 h^−1^. It is worth noting that a perfectly linear growth of *M*_n_ with increasing monomer conversion was observed while an extremely narrow dispersity remained (*Đ* = 1.04~1.11, Table S1, Figure S1), indicative of controlled polymerization with a living characteristic. Moreover, the MALDI-TOF MS spectrum of the PMMA oligomer produced by Al(BHT)_2_Me/^(Ph)Et^NHC showed only one series of mass ions (Figure 2). A plot of *m*/*z* values of this series vs. the number of monomer repeat units (x) yielded a straight line with a slope of 100.0 (the mass of MMA) and an intercept of 277.6 corresponding to ^(Ph)Et^NHC/H chain ends, supporting that the polymerization is initiated by ^(Ph)Et^NHC and proceeds through a conjugate–addition mechanism. Note that a six-membered lactone chain end caused by backbiting termination was undetectable in the MALDI-TOF MS spectrum, demonstrating the successful suppression of backbiting termination by introducing sterically hindered Al(BHT)_2_Me LA as anticipated, thus giving rise to the controlled polymerization with a living characteristic.

When the amount of Al(BHT)_2_Me was increased to 3 equivalent relative to ^(Ph)Et^NHC, the I^*^ and TOF values of the polymerization further enhanced to 60.6% and 3000 h^−1^, respectively, affording the polymer with a narrow *Đ* value of 1.13 (Table 1, Run 6). Changing the solvent from toluene to more polar DCM decreased both the activity and the I^*^ (Table 1, Run 7), as NHCs are known to decompose in DCM over time. In addition, polymerization by the other LAs in conjunction with ^(Ph)Et^NHC LB, has also been studied. The polymerization became sluggish and uncontrolled when replacing Al(BHT)_2_Me with B(C_6_F_5_)_3_ (TOF = 79.2 h^−1^), producing the polymer with broader dispersity (*Đ* = 1.48), though the I^*^ of the polymerization was higher under the same conditions (Table 1, Run 8 vs. 3). No polymerization occurred within 24 h when the strong Lewis-acidic Al(C_6_F_5_)_3_ was used as an LA for MMA polymerization (Table 1, Run 9). Switching to the alkyl aluminums, AlMe_3_ and AlEt_3_ with weak Lewis acidity and less steric hindrance were inactive for LPP up to 24 h (Table 1, Runs 10–11), while the polymerization by more sterically demanding Al*^i^*Bu_3_ exhibited very low activity (TOF = 4.1 h^−1^) and an I^*^ of 9.70% (Table 1, Run 12), producing the polymer with broad dispersity (*Đ* = 1.47). These results indicated the unicity of the Al(BHT)_2_Me-based LA when coupled with the ^(Ph)Et^NHC LB for highly active and controlled polymerization of MMA.

**Figure 3:**
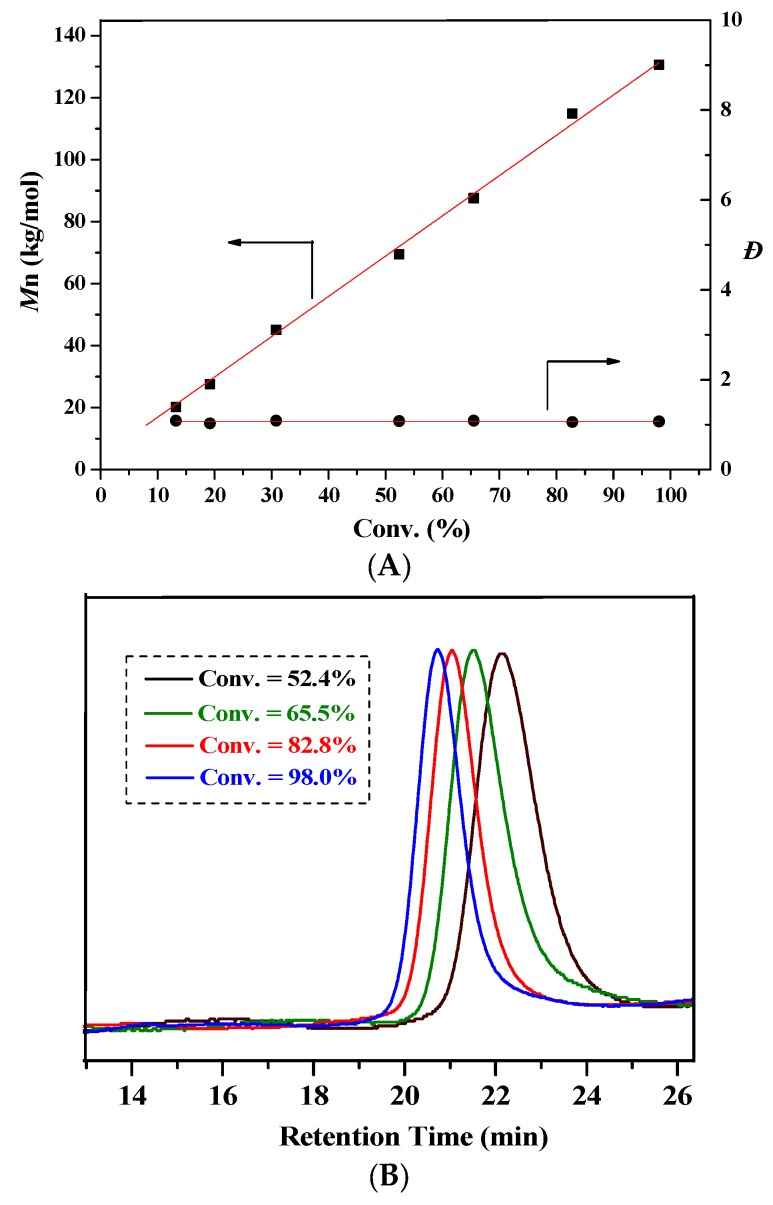
(**A**) Plots of *M*_n_ and *Đ* vs. monomer conversion (%) for the MMA polymerization by *^i^*^Pr^NHC/Al(BHT)_2_Me. (**B**) GPC traces for PMMAs produced at different monomer conversions.

Besides ^(Ph)Et^NHC, two other NHCs, *^i^*^Pr^NHC and *^t^*^Bu^NHC, were also utilized as LBs to examine the effect of the NHC structure on Al(BHT)_2_Me-based LPP. As shown in Figure 1, the monomer conversions in both LP systems also increase linearly with polymerization time after an initial induction period (*^i^*^Pr^NHC: 0.3 min, *^t^*^Bu^NHC: ca. 0 min). The activities and I^*^ values of the polymerizations follow the order of *^t^*^Bu^NHC (411 h^−1^, 9%) < *^i^*^Pr^NHC (980 h^−1^, 38%) < ^(Ph)Et^NHC (1875 h^−1^, 50%) (Table 1, Run 5 vs. 13 vs. 14). The most nucleophilic *^t^*^Bu^NHC as an LB for LPP exhibited the lowest activity and I^*^, probably due to the extremely high steric stress between the bulky *^t^*^Bu^NHC and steric demanding MMA→Al(BHT)_2_Me adduct that prevents the formation of zwitterionic species. On the other hand, compared to ^(Ph)Et^NHC, less sterically hindered *^i^*^Pr^NHC-based LP exhibited lower activity and I^*^, presumably caused by the formation of a more stable CLP, which is more difficult to dissociate so as to generate free NHC for initiating polymerization. Hence, it appears that the steric hindrance of ^(Ph)Et^NHC has a fine balance between the ability to attack the monomer and the propensity to dissociate from the CLP, thus enabling the highest activity and I^*^ in ^(Ph)Et^NHC/Al(BHT)_2_Me-mediated polymerization. It is noteworthy that the polymerization by *^i^*^Pr^NHC/Al(BHT)_2_Me is also very controlled with a living characteristic, similar to the ^(Ph)Et^NHC/Al(BHT)_2_Me system, as confirmed by a linear growth of *M*_n_ with increasing monomer conversion as well as maintained extremely narrow dispersity (*Đ*: 1.06~1.13, Figure 3, Table S2). Different from ^(Ph)Et^NHC and*^i^*^Pr^NHC-based LP systems, *^t^*^Bu^NHC/Al(BHT)_2_Me promoted uncontrolled polymerization, affording the polymer with a bimodal molecular weight distribution (Figure S2). In terms of the stereoselectivity of these LPP systems, the use of the sterically demanding Al(BHT)_2_Me LA did not noticeably affect the stereoselectivity, and the PMMAs produced at RT were typically syndio-rich (~70% rr).

Following a successful polymerization protocol developed for MMA polymerization, the ^(Ph)Et^NHC/Al(BHT)_2_Me LP system was also found to be highly active and controlled for the polymerization of the sterically encumbered *^n^*BuMA (TOF = 1500 h^−1^) and biorenewable γMMBL (TOF = 750 h^−1^), showing moderate I^*^ values (41.5~55.0%) and producing polymers with narrow dispersities (1.04~1.18), though their activities were lower than those of MMA polymerization (Table 1, Run 15 vs. 16 vs. 5). Moreover, the living characteristic of LPP by ^(Ph)Et^NHC/Al(BHT)_2_Me enabled the synthesis of well-defined diblock copolymers. The sequential addition as a result of polymerizing MMA first with MMA/Al(BHT)_2_Me/^(Ph)Et^NHC = 400/1/2 without quenching and subsequently with another 250 equivalent of *^n^*BuMA afforded the successfully well-defined diblock copolymer PMMA-*b*-P*^n^*BuMA (Table 1, Run 17), as evidenced by GPC traces (Figure S3), where PMMA produced first (*M*_n_ = 87.4 kg/mol, *Đ* = 1.13) shifted to a higher molecular weight region while the narrow dispersity remained (*M*_n_ = 105.5 kg/mol, *Đ* = 1.09).

## 3. Experimental Section

### 3.1. Materials, Reagents, and Methods

All synthesis and manipulations of air- and moisture-sensitive materials were carried out in flamed Schlenk-type glassware on a dual-manifold Schlenk line, on a high-vacuum line, or in an argon-filled glovebox. HPLC-grade organic solvents were first sparged extensively with nitrogen as they were placed into 20 L solvent reservoirs and then dried via passage through activated alumina (for dichloromethane (DCM)) and then via passage through Q-5 supported copper catalyst stainless steel columns [for toluene (TOL)]. Methyl methacrylate (MMA) was purchased from Aldrich Chemical Co. (Shanghai, China), while butyl methacrylate (*^n^*BuMA) and γ-methyl-α-methylene-γ-butyrolactone (γMMBL) were purchased from TCI Shanghai. These monomers were first degassed and dried over CaH_2_ overnight, followed by vacuum distillation. MMA was further purified by titration with neat tri(*n*-octyl)aluminum to a yellow end point and distillation under reduced pressure [38]. The purified monomers were stored in brown bottles inside a glovebox freezer at −30 °C for further use. Triisobutylaluminum (Al*^i^*Bu, 1.0 M in hexanes) was purchased from *J&K* Chemical Co. (Shanghai, China), while trimethylaluminum (AlMe_3_, 1.0 M in heptane) and triethylaluminum (AlEt_3_, 0.6 M in heptane) were purchased from Acros Chemical Co. (Shanghai, China), which were all used as received*.* 2,6-di-*tert*-Butyl-4-methylphenol (BHT-H) was purchased from Macklin and recrystallized from hexanes prior to use. Literature procedures were used to prepare MeAl(BHT)_2_ [39]. Tris(pentafluorophenyl) borane (B(C_6_F_5_)_3_) was purchased from Aldrich Chemical Co. and further purified via sublimation under vacuum. Tris(pentafluorophenyl)alane (Al(C_6_F_5_)_3_), as a (toluene)_0.5_ adduct, was prepared by ligand exchange reaction between B(C_6_F_5_)_3_ and AlMe_3_ [40]. *N*-Heterocyclic carbenes (NHCs), including 1,3-bis-(1-phenylethyl)imidazolin-2-ylidene (^(Ph)Et^NHC) [41] and 1,3-diisopropal-4,5-dimethylimidazol-2-ylidene (*^i^*^Pr^NHC) [42], were synthesized according to literature procedures, while 1,3-di-*tert*-butylimidazol-2-ylidene (*^t^*^Bu^NHC) was purchased from TCI Shanghai. Benzene-*d*_6_ was dried over a sodium/potassium alloy and vacuum-distilled or filtered.

### 3.2. Stoichiometric Reaction of Al(BHT)_2_Me with MMA and In Situ Generation of MMA→Al(BHT)_2_Me Adduct

To a benzene-*d*_6_ (0.5 mL) solution of MMA (3.5 mg, 0.035 mmol) in a J-Young NMR tube, Al(BHT)_2_Me (16.8 mg, 0.035 mmol) was added. The mixture was allowed to react for 10 min before ^1^H-NMR measurement, which indicated the formation of MMA→Al(BHT)_2_Me adduct. ^1^H-NMR (400 MHz, benzene-*d*_6_, 25 °C): *δ* 7.18 (s, 4H, Ar–*H*), 6.20 (s, 1H, =C*H*_2_), 5.11 (s, 1H, =C*H*_2_), 3.17 (s, 3H, –OC*H*_3_), 2.32 (s, 6H, Ar–C*H*_3_), 1.56 (s, 36H, –*^t^*Bu), 1.39 (s, 3H, –CH_3_), 0.01 (s, 3H, Al–C*H*_3_).

### 3.3. Stoichiometric Reaction of Al(BHT)_2_Me with (Ph)EtNHC and In Situ Generation of (Ph)EtNHC→Al(BHT)_2_Me Adduct

To a benzene-*d*_6_ (0.5 mL) solution of ^(Ph)Et^NHC (5.0 mg, 0.018 mmol) in a J-Young NMR tube, Al(BHT)_2_Me (8.7 mg, 0.018 mmol) was added. The mixture was allowed to react for 10 min before ^1^H-NMR measurement, which indicated the formation of ^(Ph)Et^NHC→Al(BHT)_2_Me adduct as two isomers A (major) and B (minor) in a 6:4 ratio. ^1^H-NMR (400 MHz, benzene-*d*_6_, 25 °C): *δ* 6.98~7.20 (m, ^(Ph)Et^NHC: 8H, Ph–*H*, Al(BHT)_2_Me: 4H, Ar–*H*), 6.61 (d, *J* = 8.0 Hz, ^(Ph)Et^NHC: 2H, Ph–*H*), 6.42 (s, 0.7H, B isomer, –C*H*=C*H*–), 6.32 (s, 1.3H, A isomer, –C*H*=C*H*–), 6.04 (q, *J* = 8.0 Hz, 0.7H, B isomer, >C*H*CH_3_), 5.98 (q, *J* = 8.0 Hz, 1.3H, A isomer, >C*H*CH_3_), 2.40 (d, *J* = 8.0 Hz, 6H, >CHC*H*_3_), 1.33~1.59 (m, 42H, –*^t^*Bu and Ar-C*H*_3_), –0.21 (s, 3H, Al–C*H*_3_).

### 3.4. General Polymerization Procedures

Polymerizations were performed either in 25 mL flame-dried Schlenk flasks interfaced to the dual-manifold Schlenk line for runs using external temperature bath or in 20 mL glass reactors inside the glovebox for ambient temperature (ca*.* 25 °C) runs. In a typical polymerization procedure, a predetermined amount of LA was first dissolved in a predetermined amount of monomer [M] and solvent inside a glovebox. The polymerization was started by the rapid addition of an LB solution in a solvent via a gastight syringe to the above-mentioned mixture containing the LA and monomer under vigorous stirring. The amount of the monomer was fixed for the varied [M]/[LB] ratio runs. After the measured time interval, a 0.2 mL aliquot was taken from the reaction mixture via syringe and quickly quenched into a 2 mL vial containing 0.4 mL of undried “wet” CDCl_3_ stabilized by 250 ppm of BHT-H; the quenched aliquots were later analyzed by ^1^H-NMR to obtain the percent monomer conversion data. After the polymerization was stirred for the stated reaction time, the polymer was immediately precipitated into 200 mL of methanol, stirred for 1 h, filtered, washed with methanol, and dried in a vacuum oven at 50 °C overnight to a constant weight.

### 3.5. Polymer Characterizations

NMR spectra were recorded on a Varian 400 MHz spectrometer. Chemical shifts for ^1^H and ^13^C spectra were referenced to internal solvent resonances and are reported as parts per million relative to SiMe_4_. Polymer number-average molecular weights (*M*_n_) and molecular weight distributions (*Đ* = *M*_w_/*M*_n_) were measured by gel permeation chromatography (GPC) analyses carried out at 40 °C and a flow rate of 1.0 mL/min, with THF as the eluent, on a Waters 1515 GPC instrument equipped with a Waters 2414 refractive-index detector and three Waters Styragel HR-3,4,5 columns connected in series. The instrument was calibrated with 10 PMMA standards, and chromatograms were processed with Waters Empower software (Version 3, Waters Ireland, Dublin, Ireland). The isolated low molecular weight samples were analyzed by matrix-assisted laser desorption/ionization time-of-flight mass spectroscopy (MALDI-TOF MS); the experiment was performed on an Shimadzu Biotech Axima Performance MALDI-TOF mass spectrometer (Kyoto, Japan) operated in positive ion reflector mode using an Nd:YAG laser at 355 nm and a 25 kV accelerating voltage. A thin layer of a 1% NaI solution was first deposited on the target plate, followed by 0.6 μL of both sample and matrix (dithranol, 10 mg/mL in 50% ACN, 0.1% TFA). External calibration was done using a peptide calibration mixture (4–6 peptides) on a spot adjacent to the sample.

## 4. Conclusions

In summary, we have established an effective strategy for controlled and efficient Lewis pair polymerizations of conjugated polar alkenes including MMA, *^n^*BuMA, and γMMBL under mild conditions. Success is attributable to the judicious selection of cooperative LAs and LBs: the introduction of sterically demanding Al(BHT)_2_Me as an LA can completely shut down intramolecular backbiting termination, and the utilization of a strong nucleophile NHC as an LB ensures high activity and acceptable initiation efficiency. Investigation into the scope of LAs showed that an Al(BHT)_2_Me-based LA is unique for such LPP, while the other less sterically hindered LAs, such as Al(C_6_F_5_)_3_, B(C_6_F_5_)_3_, AlMe_3_, AlEt_3_, and Al*^i^*Bu_3_, did not lead to any polymer formation or resulted in uncontrolled/low active polymerization. In this study, compared to the *^i^*^Pr^NHC and*^t^*^Bu^NHC, the ^(Ph)Et^NHC-based LP coupled with Al(BHT)_2_Me, which showed a fine balance between the ability to attack monomers and the propensity to dissociate from CLPs, exhibited the highest activity (TOF up to 3000 h^−1^) and an initiation efficiency up to 60.6%. Note that the ^(Ph)Et^NHC/Al(BHT)_2_Me and *^i^*^Pr^NHC/Al(BHT)_2_Me LP systems have good control over the polymerization of MMA with a living characteristic, as evidenced by chain-extension experiments and chain-end analysis. The controlled characteristic can also be extended to *^n^*BuMA and MMBL polymerizations and enable the synthesis of well-defined diblock copolymers.

## Figures and Tables

**Table 1:** Selected results of polymerizations by Al(BHT)_2_Me/NHC LPs ^1^.

Run	LB	LA	Monomer (M)	M/LA/LB	Solvent	Time (min)	Conv. (%) ^2^	*M*_n_^3^ (kg/mol)	*Đ* ^3^	I^*^^4^ (%)
1	**1**	-	MMA	100/-/1	TOL	1440	0	-	-	-
2	-	Al(BHT)_2_Me	MMA	100/1/-	TOL	1440	0	-	-	-
3	**1**	Al(BHT)_2_Me	MMA	100/2/1	TOL	3	100	54.1	1.11	19.0
4 ^5^	**1**	Al(BHT)_2_Me	MMA	100/2/1	TOL	30	100	343.2	1.14	3.00
5	**1**	Al(BHT)_2_Me	MMA	500/2/1	TOL	16	100	102.7	1.06	50.0
6	**1**	Al(BHT)_2_Me	MMA	500/3/1	TOL	10	100	82.5	1.13	60.6
7	**1**	Al(BHT)_2_Me	MMA	500/2/1	DCM	25	100	127.9	1.07	39.1
8	**1**	B(C_6_F_5_)_3_	MMA	100/2/1	TOL	75	99	15.8	1.48	65.2
9	**1**	Al(C_6_F_5_)_3_	MMA	100/2/1	TOL	1440	0	-	-	-
10	**1**	AlMe_3_	MMA	100/2/1	TOL	1440	0	-	-	-
11	**1**	AlEt_3_	MMA	100/2/1	TOL	1440	19.6	-	-	-
12	**1**	Al*^i^*Bu_3_	MMA	100/2/1	TOL	1440	99.8	106	1.47	9.70
13	**2**	Al(BHT)_2_Me	MMA	500/2/1	TOL	30	98	130.6	1.07	38.0
14	**3**	Al(BHT)_2_Me	MMA	250/2/1	TOL	34	93.2	258.0 ^6^	1.08 ^6^	9.03
15	**1**	Al(BHT)_2_Me	*^n^*BuMA	500/2/1	TOL	20	100	128.9	1.18	55.0
16	**1**	Al(BHT)_2_Me	MMBL	500/2/1	DCM+ TOL ^7^	40	98.5	133.0	1.04	41.5
17 ^8^	**1**	Al(BHT)_2_Me	MMA/*^n^*BuMA	(400 + 250)/2/1	TOL	16 10	100	87.4 105.5	1.13 1.09	-

^1^ Conditions: M/LA/LB = 100/2/1: LB = 37.4 umol, [MMA] = 1.56 M, M/LA/LB = 500/2/1: LB = 18.7 umol, [MMA] = 0.94 M, 250/2/1: LB = 37.4 umol, [MMA] = 0.94 M. ^2^ Monomer conversions were measured by ^1^H NMR spectra. ^3^ Number-average molecular weight (*M*_n_) and dispersity (*Đ* = *M*_w_*/M*_n_) were determined by GPC at 40 °C in THF relative to PMMA standards. ^4^ Initiation efficiency (I^*^) = *M*_n_(calcd.)/*M*_n_(exptl), where *M*_n_(calcd.) = MW(M) × [M]/[LB] × conversion (%) + MW(chain-end groups). ^5^ The polymerization was carried out by premixing LA and LB first followed by adding MMA. ^6^ GPC trace showed a small (ca. 9%) high MW shoulder peak. ^7^ The polymerization was started by the addition of an NHC solution in toluene to DCM solution of γMMBL and Al(BHT)_2_Me. ^8^ Diblock copolymer was prepared by initiating MMA block first followed by adding *^n^*BuMA.

## Supplementary Materials

Supplementary materials are available online.
