# Supplementary material for: Controlled and Efficient Polymerization of Conjugated Polar Alkenes by Lewis Pairs Based on Sterically Hindered Aryloxide-Substituted Alkylaluminum
Source: Molecules. 2018 Feb 17;23(2):442. doi: 10.3390/molecules23020442 (PMC6017945; doi:10.3390/molecules23020442)
Supplement: Supplementary file 1 [file molecules-23-00442-s001.pdf]

Supplementary Information

Article

Controlled and Efficient Polymerization of Conjugated Polar Alkenes by Lewis Pairs Based on Sterically Hindered Aryloxide-Substituted Alkylaluminumitle

Xiaojun Wang [1], Yixin Zhang [1] and Miao Hong [1,*]

[1] State Key Laboratory of Organometallic Chemistry, Center for Excellence in Molecular Synthesis, Shanghai Institute of Organic Chemistry, University of Chinese Academy of Sciences, Chinese Academy of Sciences, 345 Lingling Road, Shanghai 200032, China; wangxiaojun@sioc.ac.cn (X.W.); zhangyixin1@sioc.ac.cn (Y.Z.)

* Correspondence: miaohong@sioc.ac.cn; Tel.: +86-2154925610

Table S1. M_n and *Đ* results of PMMAs produced by $Al(BHT)_2Me/^{(Ph)Et}NHC$.[a] .

Time (min)	Conv.(%)	M_n (kg/mol)	*Đ*
4	17.6	22.4	1.11
6	33.3	43.1	1.04
8	46.4	52.9	1.10
10	62.4	69.6	1.05
16	100	102.7	1.06

[a] Conditions: MMA/$Al(BHT)_2Me/^{(Ph)Et}NHC$ = 500/2/1.

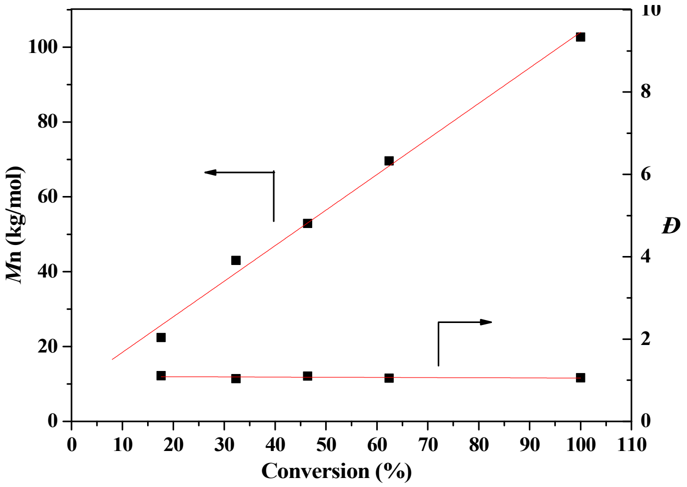

Figure S1. Plots of M_n and *Đ* vs monomer conversion (%) for the MMA polymerization by $^{(Ph)Et}NHC/Al(BHT)_2Me$.

Table S2. M_n and $Đ$ results of PMMAs produced by Al(BHT)$_2$Me/^{iPr}NHC.[a] .

Time (min)	Conv.(%)	M_n (kg/mol)	$Đ$
4	13.2	20.2	1.09
6	19.2	27.6	1.13
10	30.8	45.1	1.09
16	52.4	69.5	1.08
20	65.5	87.6	1.09
25	82.8	114.8	1.06
30	98.0	130.6	1.07

[a] Conditions: MMA/Al(BHT)$_2$Me/^{iPr}NHC = 500/2/1.

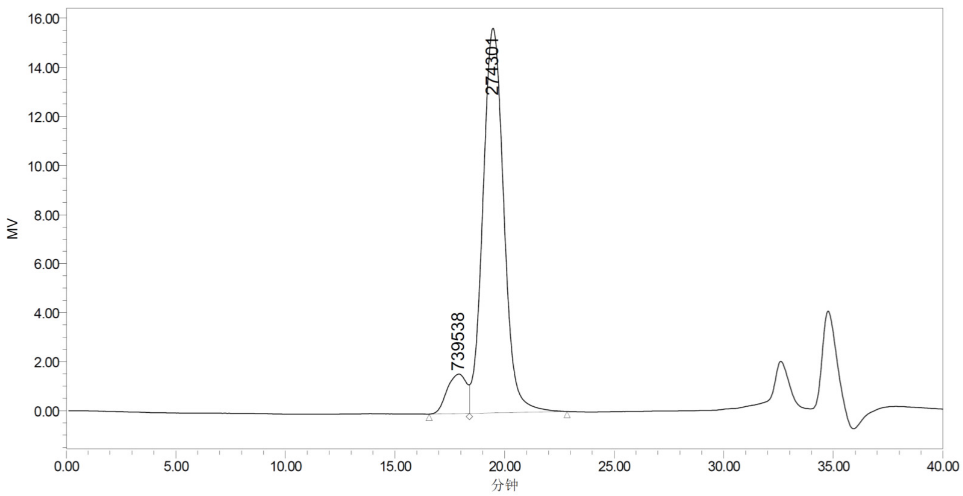

Figure S2. GPC trace of PMMA produced by Al(BHT)$_2$Me/^{tBu}NHC (Table1, Run 14): M_n = 258.0 kg/mol, $Đ$ = 1.08 (91%); M_n = 786.0 kg/mol, $Đ$ = 1.07 (9%).

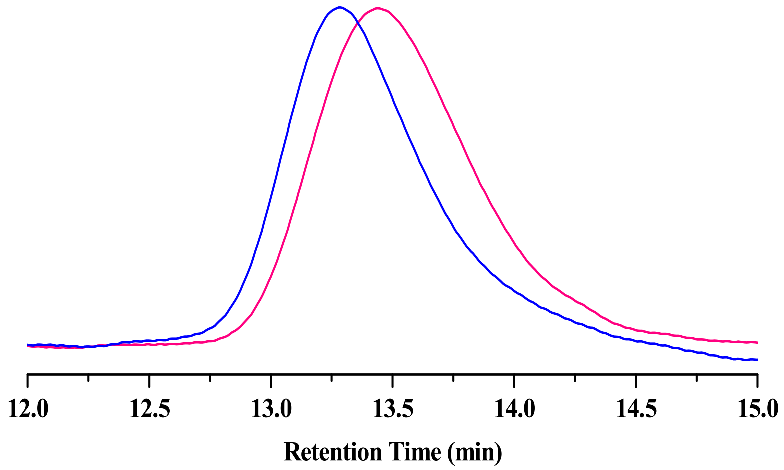

Figure S3. GPC trace of PMMA (red) and PMMA-*b*-P^{n}BuMA (blue) produced by Al(BHT)$_2$Me/$^{(Ph)Et}$NHC [M_n and $Đ$ were measured by GPC analyses carried out at 40 ºC and a flow rate of 1.0 mL/min, with DMF as the eluent, on a Waters 2695 GPC instrument equipped with a OPTILAB® T-rEX Interferometric Refractometer detector and PLgel 5 μm guard and two PLgel 5 μm mixed-C columns (Agilent, linear range of molecular weight = 200–2,000,000) connected in series. The instrument was calibrated with 10 PMMA standards, and chromatograms were processed with OPTILAB software].

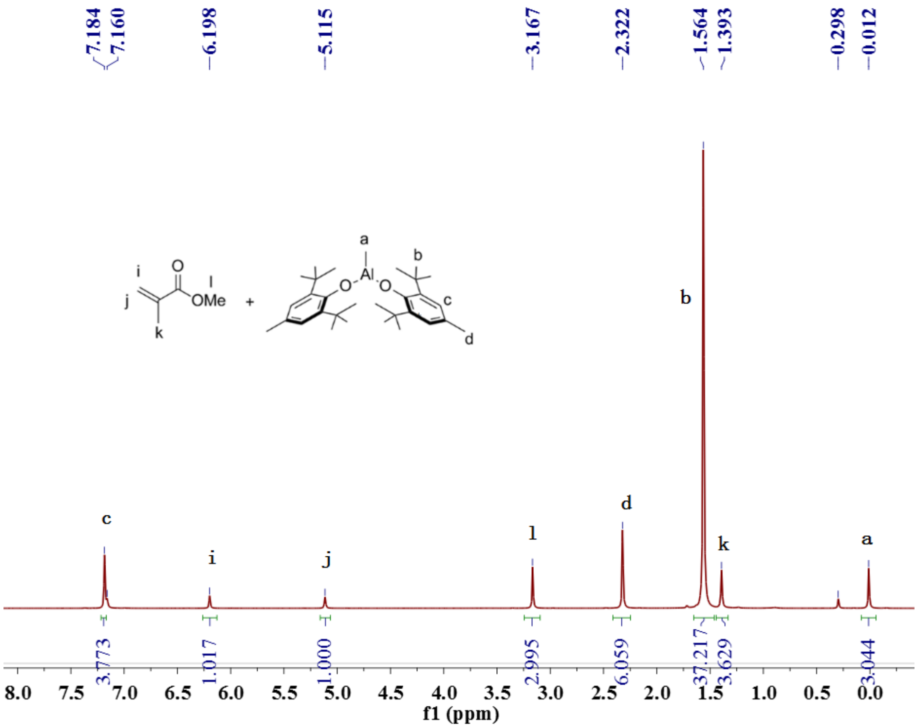

Figure S4. ^{1}H-NMR spectrum of MMA→Al(BHT)$_2$Me adduct in benzene-d_6.

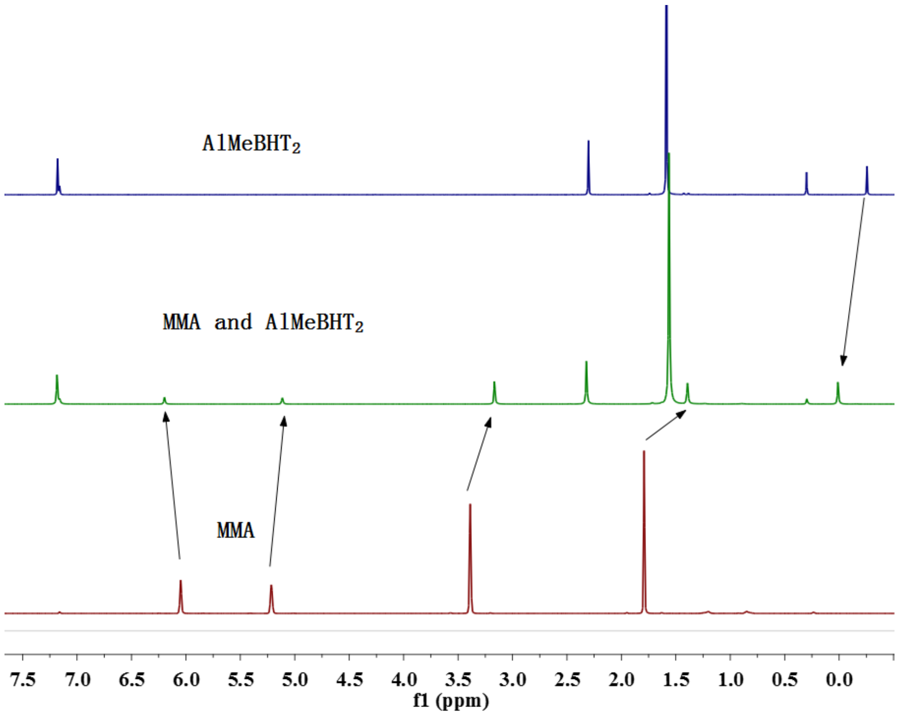

Figure S5. Comparison of ^{1}H NMR spectra in benzene-d_6: (blue) Al(BHT)$_2$Me, (green) MMA→Al(BHT)$_2$Me adduct, (red) MMA.

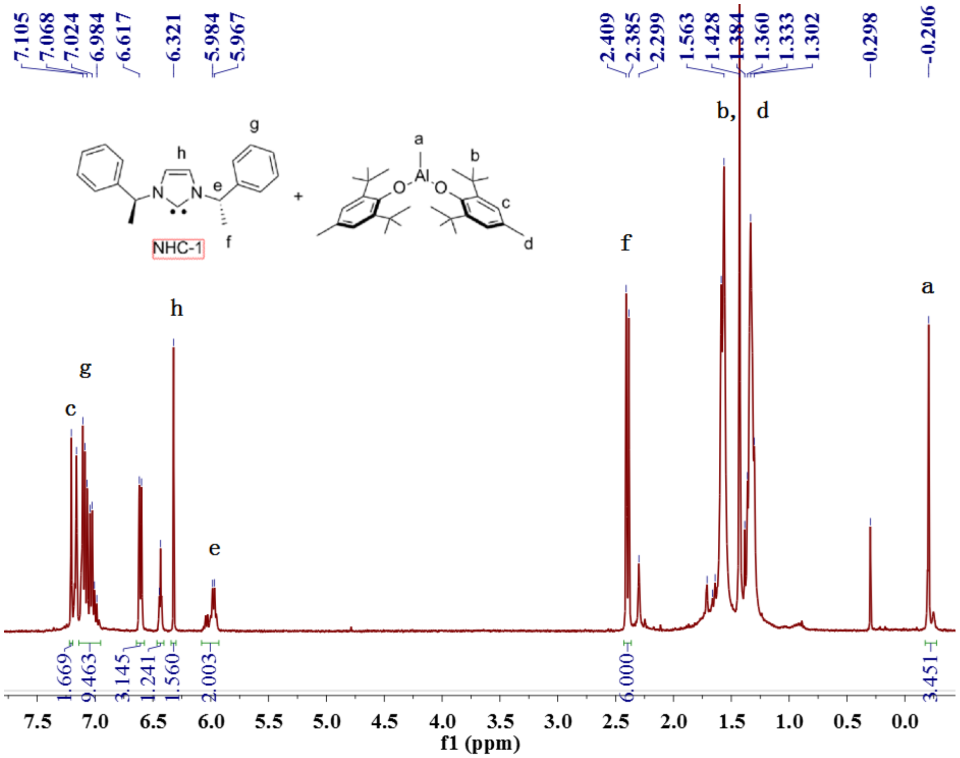

Figure S6. ^{1}H-NMR spectrum of $^{(Ph)Et}$NHC→Al(BHT)$_2$Me adduct in benzene-d_6.

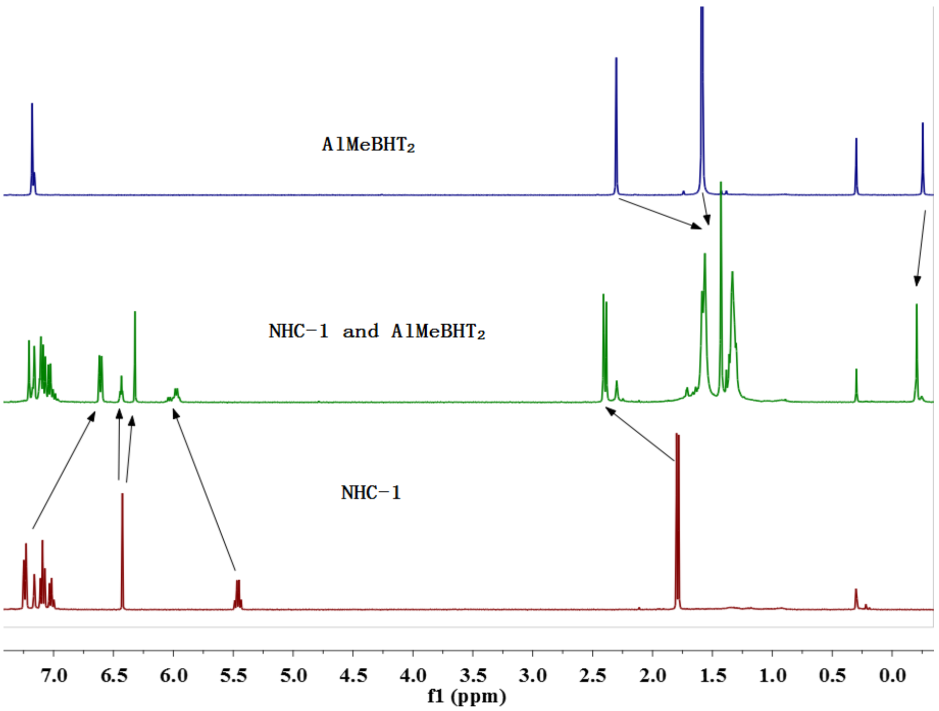

Figure S7. Comparison of ^{1}H NMR spectra in benzene-d_6: (blue) Al(BHT)$_2$Me, (green) $^{(Ph)Et}$NHC→Al(BHT)$_2$Me adduct, (red) $^{(Ph)Et}$NHC.

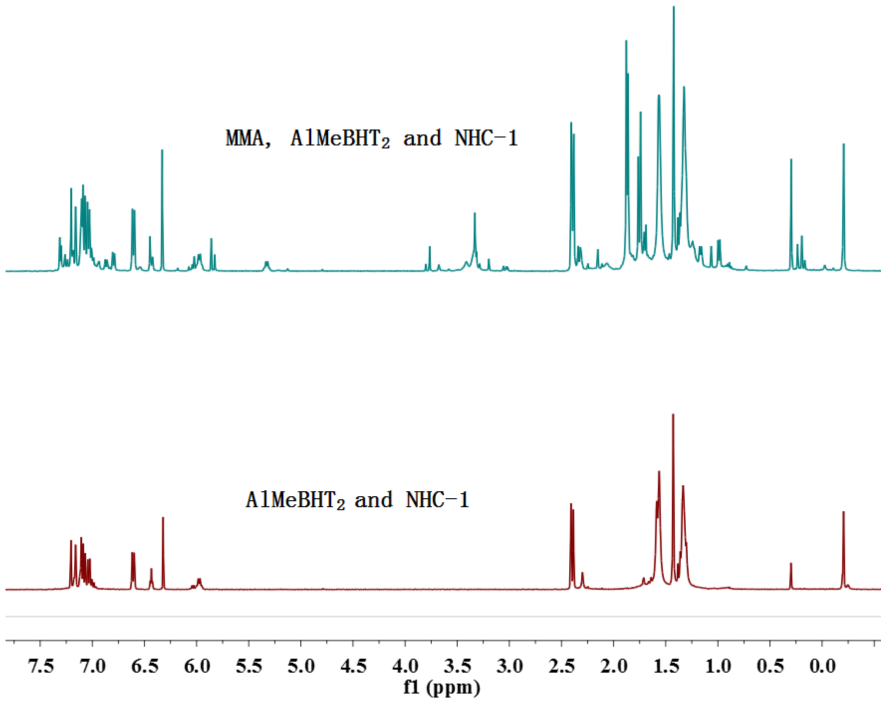

Figure S8. Comparison of ^{1}H-NMR spectra in benzene-d$_6$: (blue) stoichiometric reaction of $Al(BHT)_2Me$, MMA, and $^{(Ph)Et}$NHC, (red) $^{(Ph)Et}$NHC→$Al(BHT)_2Me$ adduct.
